# How Much do Needlestick Injuries Cost? A Systematic Review of the Economic Evaluations of Needlestick and Sharps Injuries Among Healthcare Personnel

**DOI:** 10.1017/ice.2016.48

**Published:** 2016-03-29

**Authors:** Alice Mannocci, Gabriella De Carli, Virginia Di Bari, Rosella Saulle, Brigid Unim, Nicola Nicolotti, Lorenzo Carbonari, Vincenzo Puro, Giuseppe La Torre

**Affiliations:** 1 Department of Public Health and Infectious Diseases, Sapienza University of Rome, Italy; 2 Department of Epidemiology, Pre-Clinical Research and Advanced Diagnostics, L. Spallanzani National Institute for Infectious Diseases, Rome, Italy; 3 Department of Economics and Finance & CEIS, University of Rome “Tor Vergata”Italy

## Abstract

**OBJECTIVE:**

To provide an overview of the economic aspects of needlestick and sharps injury (NSI) management among healthcare personnel (HCP) within a Health Technology Assessment project to evaluate the impact of safety-engineered devices on health care

**METHODS:**

A systematic review of economic analyses related to NSIs was performed in accordance with the PRISMA statement and by searching PubMed and Scopus databases (January 1997–February 2015). Mean costs were stratified by study approach (modeling or data driven) and type of cost (direct or indirect). Costs were evaluated using the CDC operative definition and converted to 2015 International US dollars (Int$).

**RESULTS:**

A total of 14 studies were retrieved: 8 data-driven studies and 6 modeling studies. Among them, 11 studies provided direct and indirect costs and 3 studies provided only direct costs. The median of the means for aggregate (direct + indirect) costs was Int$747 (range, Int$199–Int$1,691). The medians of the means for disaggregated costs were Int$425 (range, Int$48–Int$1,516) for direct costs (9 studies) and Int$322 (range, Int$152–Int$413) for indirect costs (6 studies). When compared with data-driven studies, modeling studies had higher disaggregated and aggregated costs, but data-driven studies showed greater variability. Indirect costs were consistent between studies, mostly referring to lost productivity, while direct costs varied widely within and between studies according to source infectivity, HCP susceptibility, and post-exposure diagnostic and prophylactic protocols. Costs of treating infections were not included, and intangible costs could equal those associated with NSI medical evaluations.

**CONCLUSIONS:**

NSIs generate significant direct, indirect, potential, and intangible costs, possibly increasing over time. Economic efforts directed at preventing occupational exposures and infections, including provision of safety-engineered devices, may be offset by the savings from a lower incidence of NSIs.

*Infect Control Hosp Epidemiol* 2016;37:635–646

Occupational exposures of healthcare personnel (HCP), especially nurses and surgeons, to bloodborne pathogens are frequent events in hospitals.^1^ In Italy every day, ~300 HCP sustain an injury involving a contaminated needle or sharp medical device (needlestick and sharps injuries, NSIs), totaling >100,000 accidents per year, but only an estimated 45% are officially reported.^2^ Similar figures are available for the United States where, before legislation mandated widespread implementation of needlestick-prevention devices incorporating a safety mechanism to cover the tip after use (safety-engineered devices, SEDs), 385,000 NSIs were estimated to occur annually, 60% of which were unreported.^3^ Available data from other European countries and all continents clearly demonstrate the high NSI burden worldwide.^4^ ^–^ ^8^

These NSIs have the potential to transmit virtually every pathogen present in human blood, either transiently or persistently.^9^ ^,^ ^10^ However, hepatitis B virus (HBV), hepatitis C virus (HCV), and human immunodeficiency virus (HIV), all bearing significant morbidity and mortality, together account for the vast majority of occupational infections reported in healthcare. In 2000, in the absence of interventions to prevent NSIs, these agents were estimated to have caused >80,000 new infections among HCP per year worldwide.^11^

Therefore, administrative, behavioral, and technical interventions aiming to reduce NSI frequency have been progressively introduced, including recommendations to prevent improper needle manipulations,^12^ education,^13^ provision of sharps containers for appropriate elimination of used devices,^14^ and SEDs.^15^ ^,^ ^16^ Each intervention has contributed to decreasing NSI frequency, but the best preventative strategy (ie, incorporating all these elements), requires significant investments of time, resources, and effort, which has limited their widespread implementation.^17^

Preventive interventions have therefore been included and have thus been reinforced in specific regulations regarding safety at work. In the United States, the Needlestick Safety and Prevention Act was passed in 2000,^18^ and the Occupational Safety and Health Administration endorsed the use of safe needles or needleless devices for the collection and/or withdrawal of body fluids and for the administration of fluids and medications.^19^ ^,^ ^20^ In Europe, the Council Directive 2010/32/EU, “Prevention from sharp injuries in the hospital and healthcare sector,” fully in force since 2013, protects HCP from NSIs and their consequences, setting up integrated policies regarding risk assessment, risk prevention, training, education, and monitoring. Among the prevention measures, SEDs must be made available based on risk assessment, whereas HBV vaccination must be universally provided free of charge. Monitoring includes investigating the causes and circumstances of the accident and immediate care for the injured HCP that includes post-exposure prophylaxis (PEP), the necessary medical tests, health surveillance, and counseling where appropriate. Additionally, medical treatment is guaranteed. The economic impact of this directive is expected to be significant.^21^

Several studies have considered the economic burden of occupational NSIs,^22^ ^–^ ^25^ mostly providing direct costs (ie, post-exposure management) and indirect costs (ie, counselling NSI victims, staff absence and compensation) from different perspectives and, more rarely, evaluating NSIs reduction after the introduction of preventive measures and the resulting economic impact. However, these studies vary widely in terms of setting, source of epidemiologic data, and considered costs, making it difficult to define a standardized cost for NSI management and prevention.

Given the increased costs of SEDs compared to conventional devices, in 2012, a Health Technology Assessment project was launched in Italy to evaluate this new technology and its overall impact on health care, including an economic analysis of costs deriving from NSIs and savings arising from their prevention. This systematic review represents the first part of this project.

## MATERIALS AND METHODS

A systematic review of economic analyses of occupational NSIs among HCP was performed in accordance with the PRISMA statement.^26^ Independently, 2 authors consulted PubMed and Scopus databases and resolved eventual discrepancies by consulting a third author. The research algorithm used the following keywords: “cost, needlestick injuries” and “cost, occupational exposures, blood injuries.” Inclusion criteria were language (English, Italian, French, or Spanish) and publication date (January 1997–February 2015).

**FIGURE 1:**
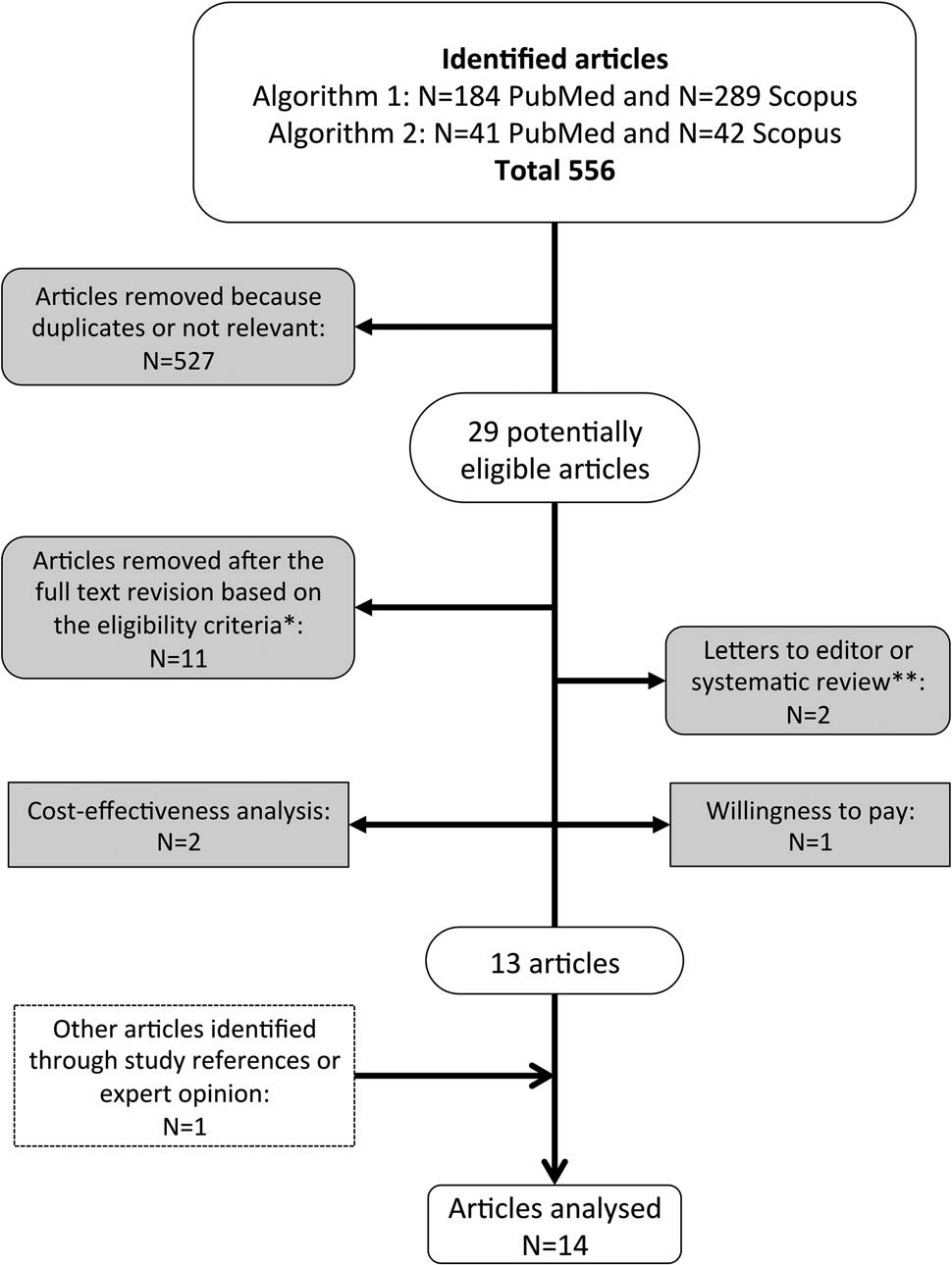
Flow-chart of the selection process. *Language other than English, Italian, French or Spanish, or published before January 1997. **Study references were examined to identify other eligible studies.

The titles and abstracts of all articles were evaluated, eliminating duplicates. Articles were then assessed for eligibility through a full-text examination, and an article was included in the analysis if it provided an original estimate of either direct or indirect costs of managing a single NSI. The main stages of the systematic review are shown in Figure 1. Categorization and organization of the reference list were performed using JabRef 2.7.2 software.

Data extraction forms included author’s name, publication year, country, source of the occurrence data, study period, population studied, NSI incidence, perspective, cost estimating approach (modeling or data-driven), items considered in cost, type of cost studied (direct and/or indirect), injury management cost (range), and currency equivalent.

To compare monetary values expressed in different currencies and for different years, the national inflation rates provided by the World Bank (http://data.worldbank.org/indicator/fp.cpi.totl.zg) were used to express the injury management costs in national currencies 2015. These values were then converted to 2015 International US dollars (Int$) using purchasing power parity (ppp) exchange rates (http://data.worldbank.org/indicator/pa.nus.ppp). To facilitate comparisons, all costs included in this review were converted to Int$.

Data were analyzed by computing mean, median, and standard deviation (SD) as well as minimum and maximum mean costs abstracted from each article. The weighted mean was used to account for the different proportion of resources used according to the type of event. The findings were stratified by study approach and type of cost (direct and/or indirect). All data used in this analysis were true cost data.

Direct and indirect costs of an NSI were evaluated using the CDC operative definitions:

1. Direct costs: Costs generally borne by a healthcare organization when an NSI occurs: baseline and follow-up laboratory testing, PEP, and other treatment eventually provided, including PEP side-effect management. According to which costs are borne by the organization, in certain circumstances, workers’ compensation or the management of occupationally exposed HCP may be included with direct costs.

2. Indirect costs: These costs include time and wages diverted to receiving or providing exposure-related care: lost productivity associated with reporting and receiving initial and follow-up treatment for the exposure; healthcare provider time to evaluate and treat an employee; and healthcare provider time to evaluate and test the source, including obtaining informed consent for testing if applicable. For occupational health and infection control practitioners, these measures are part of their job responsibilities and therefore are not considered a diversion. Moreover, when staff absence and compensation are borne by a third-party payer, these may not be included with indirect costs.^27^

When analyzing in detail the items included within direct and indirect costs in each study, the authors originally assigned baseline and follow-up post-exposure visits differently according to the study context. However, when stratifying the study findings, the post-exposure visits, when disaggregated, were always considered as a direct cost and were assigned accordingly.

The quality of each study was evaluated using Drummond’s checklist modified by La Torre et al.^28^

## RESULTS

In total, 14 studies from Europe, America, Asia, and Australia published from 1997 to 2013 met all the inclusion criteria.^8^ ^,^ ^17^ ^,^ ^22^ ^,^ ^24^ ^,^ ^25^ ^,^ ^29^ ^–^ ^37^ Their characteristics are reported in Tables 1 and 2.

**TABLE 1:** Characteristics of the Studies of Economic Analysis of Occupational Needlestick and Sharps Injuries (NSIs) Among Healthcare Personnel (HCP) Included in the Systematic Review, 1997–2013

Country	Source of Occurrence Data	Study Period	Population	Baseline NSI Incidence With Conventional Devices^a^	NSI Incidence After Intervention^a^	Study (Reference)
United States	Longitudinal surveillance	2 y (June 1995–May 1997)	HCP (hospital A: CH; hospital B: UH)	Hospital A: 38.3/100 beds per year; hospital B: 66/100 beds per year	Not applicable	33
Spain	Longitudinal surveillance	18 mo (January 1993–August 1995)	UH HCP	8.2/100 HCP per year	Not applicable	37
France	Longitudinal surveillance	Baseline: 1 y (1990); after: 3 y (1995–1997)	UH HCP	12.7/100,000 needles used	6.4/100,000 needles used (education seminars+SEDs)	17
France	Longitudinal surveillance	1 y (2000)	UH HCP	4.1/100 HCP per year	Not applicable	36
United States	Retrospective survey (recall)	1 y (August 2003–August 2004)	CH nurses	44.8/100 nurses per year	Not applicable	34
Spain	Longitudinal surveillance	5 y (January 1998–December 2002)	UH HCP	7.7/100 HCP per year	Not applicable	22
Italy	Longitudinal surveillance and systematic review	75% from national longitudinal surveillance (2000–2003); 25% from systematic review of literature (2002–2005)	Hospital HCP	Hospital A: 7.8/100,000 needles used per year; hospital B: 18.4/100,000 needles used per year.	Hospital A: 0.9/100,000 needles used per year; hospital B: 2.2/100,000 needles used per year (SEDs)	30
Spain	Longitudinal surveillance	1 y (March 2002–Februay 2003)	UH HCP	19.4/100 beds per year	7.2/100 beds per year (SEDs);	29
					3.2/100 beds per year (SEDs+education and/or change in procedure)	
United States	Estimates from literature data	1 y (1997–1998)	Hospital and non-hospital HCP	0.7/100 FTE in non-hospital HCP; 1.6/100 FTE in hospital HCP	Not applicable	35
United States	Opportunistic sample from longitudinal surveillance	1 y (2003)	HCP	Not applicable	Not applicable	24
Sweden	Longitudinal surveillance	1 y (2002)	HCP	3.14/100 FTE per year (1.89 with hollow-bore needles; 60% of the total)	1.1/100 FTE per year (SEDs)	31
Chile	Longitudinal surveillance	5 y (2003–2007)	University healthcare students	0.9/100 students per year	Not applicable	8
Belgium	Estimates from literature and market data	1 y (1999–2000)	HCP	Injection needles used 10.8/100,000;	Injection needles used 1.5/100,000;	32
				infusion therapy 26.0;	infusion therapy 8.08;	
				insulin therapy 23.5;	insulin therapy 3.3;	
				blood collection 23.4	blood collection 7.0 (SEDs)	
Korea	Longitudinal surveillance	4 mo (October 2005–February 2006)	CH HCP	2.9/100 FTE per year or 6.1/100 beds per year	Not considered	25

NOTE. CH, community hospital; FTE, full-time equivalent; HCP, healthcare personnel (doctors, nurses, technicians, students, etc.); NSIs, needlestick and sharps injuries; UH, university hospital; SEDs, safety-engineered devices.

a NSI incidence rates were standardized according to the available denominator (100 beds; 100 FTE or HCP; 100,000 used devices).

**TABLE 2:** Economic Characteristics of the Studies of Economic Analysis on Occupational Needlestick and Sharps Injuries Among Healthcare Personnel Included in the Systematic Review, 1997–2013 (Temporal Publication Order)

Perspective	Approach	Determinants of Cost	Type of Cost^a^	Costs According to the CDC Definition^b^	Weighted Mean Cost per Injury, Int$ (Range)		Currency/Year	Value per Injury, Int$ (Range)^c^		Comments on Economic Results	Study (Reference)
NHS	Data-driven	Laboratory tests, prophylaxis, lost productivity (exposed HCP and visiting staff)	Direct/indirect	Yes	Direct+indirect Direct Indirect	605 (197–1,094) 473 (NC) 250 (NC)	US$/1997	Direct+indirect Direct Indirect	893 (290–1,614) 698 369	Hospital A: $672 (range: $340-1025); Hospital B: $539 (range: $197–1,094)	33
Third party (Social Security)	Modeling	Laboratory tests, prophylaxis, medical visits, lost productivity (exposed HCP, visiting and administration staff), overhead	Direct+indirect	…	Direct+indirect	39,564 (23,074–86,864)	Pesetas/1994	Direct+indirect	648 (378–1,423)		37
Hospital	Data driven	Laboratory tests, prophylaxis (including 7 d off work), medical visits, lost productivity (exposed HCP and visiting staff)	Direct+indirect	…	Direct+indirect	325 (NC)	US$/1998	Direct+indirect	317	The study includes (separately) the costs of measures taken to reduce injury rates. The cost-effectiveness is $4,000 per injury prevented.	17
Hospital	Data driven	Laboratory tests, prophylaxis, medical visits, lost productivity (exposed HCP)	Direct/ indirect	No	Direct+indirect Direct Indirect	1,121 1,005 (NC) 116 (NC)	Euro/2000	Direct+indirect Direct Indirect	1,691 1,516 175	Total occupational exposures cost in 1 year: €68,310; mean = €281; median = €250;	36
Societal	Data driven	Laboratory tests, treatment, medical visits, medication, lost productivity (exposed HCP and visiting staff), management of HIV PEP side effects, emotional distress and anxiety	Direct/indirect	No	Direct+indirect Direct Indirect	159 (145–201) 38 (37–39) 121 (107–163)	US$/2004	Direct+indirect Direct Indirect	199 (182–252) 48 (46–49) 152 (134–204)	NSI management annual cost: range: $25,896–$36,066. Direct+indirect costs per injured nurse: mean = $259; (range, $235–$328); direct costs per injured nurse: mean = $113 (range, $89.7–$182.2); indirect costs: mean = $146.	34
NHS	Modeling	Laboratory tests, treatment, medical visits, medical instruments; lost productivity (exposed HCP and visiting staff), overhead	Direct+indirect	NA	Direct+indirect	388 (172–1,502)	Euro/ 2002	Direct+indirect	714 (317–2,765)		22
Hospital and NHS	Modeling	Laboratory tests, treatment, medical visits, lost productivity (exposed HCP and visiting staff)	Direct/ indirect	Yes	Direct+indirect Direct Indirect	850 (750–1,320) 586 (516–910) 265 (233–411)	Euro/2005	Direct+indirect Direct Indirect	1,324 (1,168–2,056) 913 (804–1,417) 413 (363–640)	NSI management annual cost: 36 million euros	30
Hospital	Data driven	Laboratory tests, treatment, nurse and medical visits	Direct+indirect	NA	Direct+indirect	220	Euro/2003	Direct+indirect	418	Cost-effectiveness for avoided injury adopting SEDs: IV catheters: €2.65; hypodermic syringes: €869.79; butterfly needles €1,195.99; needleless administration sets €4,954.55; short IV catheters: €31,563.91.	29
Societal	Modeling	Laboratory tests, treatment, medical visits, lost productivity (exposed HCP)	Direct/indirect	Yes	Direct+indirect Direct Indirect	596 339 257	US$/2004	Direct+indirect Direct Indirect	747 425 322	National annual costs: tests: $103,125,746; lost productivity: $81,187,457; subsequent infections: $4,186,548 Total cost for injuries: $188,499,751	35
Hospital	Data driven	Laboratory tests, treatment, lost productivity (exposed HCP and visiting staff), additional wages for management of HIV PEP side effects	Direct+indirect	NA	Direct+indirect	1,161 (71–4,838)	US$/2003	Direct+indirect	1,470 (90–6,127)	Mean cost related to the infectious status of the source:-$2,456 if positive for HIV (HBV, HCV included); $650 if positive for HCV; $376 for not infected/unknown source	24
NHS	Modeling	Laboratory tests, treatment, medical visits	Direct	Yes	Direct	2,513	Swedish kronor (SEK)/2007	Direct	294	Adopting safety devices reduces annual cost by €843,426.	31
University (training school)	Data driven	Laboratory tests; treatment; medical visits	Direct	Yes	Direct	149	US$/2007	Direct	170		8
Hospital	Modeling	Laboratory tests, treatment, medical visits, lost productivity (exposed HCP and visiting staff), compensation and litigation	Direct/indirect	No	Direct+indirect Direct Indirect	867 (273–2,060) 617 (250–1,189) 250 (23–606)	Euro/2012	Direct+indirect Direct Indirect	1,049 (331–2,493) 747 (303–1,439) 303 (28–734)	Direct costs: €210.01 (low-risk injuries, 61% of exposures) and €950.34 (high-risk injuries, 39%) Indirect costs: €63.22 (low risk) and €844.22 (high risk)	32
Hospital	Data driven	Laboratory tests, treatment, medical visits, surgical treatment, lost productivity (exposed HCP)	Direct	Yes	Direct	119,673 Korean won ($125)	Korean won and US dollar (955:1)/2006	Direct	173	Laboratory tests account for 52.6% of the cost (45.9% for the HCP). Mean NSI management cost is higher in Seoul ($139) vs the suburbs ($80). Regarding indirect costs, there were no lost working days among the exposed HCP in the study.	25

NOTE. Int$, 2015 International US dollars; HBV, hepatitis B virus; HCP, healthcare personnel; HCV, hepatitis C virus; HIV, human immunodeficiency virus; i.v., intravenous; NA, not applicable; NC, not computable; NHS, National Health System; NSI, needlestick and sharps injury; PEP: post-exposure prophylaxis; SEDs, safety-engineered devices.

a Direct+indirect: aggregated costs; direct/indirect: disaggregated costs.

b Data on categorization of the costs adapted according to Centers for Disease Control (CDC): direct costs include laboratory tests, treatment, medical visits; indirect costs include lost productivity, time-off productivity.

c Injury management costs were expressed in national currencies of 2015 using the national inflation rates provided by the World Bank (http://data.worldbank.org/indicator/fp.cpi.totl.zg), then these were converted to 2015 International US$ (Int$) using purchasing power parity (ppp) exchange rates (http://data.worldbank.org/indicator/pa.nus.ppp).

### Cost Analysis

Of the 14 studies estimating costs, 8 studies were data driven, while 6 studies used modeling (Table 3); 11 studies provided both direct and indirect costs (aggregated or disaggregated), while 3 studies only analyzed direct costs (Tables 2 and 3).

**TABLE 3:** Distribution of Studies of Economic Analysis on Occupational Needlestick and Sharps Injuries Among Healthcare Personnel According to Type of Provided Costs and Study Approach

		Type of Approach	
No. of studies	Type of Cost	Data Driven	Modeling
3	Direct costs	2	1
6	Direct/indirect costs (disaggregated)	3	3
5	Direct+indirect costs (aggregated)	3	2
14	Total	8	6

Costs were analyzed mainly from a hospital or university perspective in 7 studies^8^ ^,^ ^17^ ^,^ ^24^ ^,^ ^25^ ^,^ ^29^ ^,^ ^32^ ^,^ ^36^; 3 studies adopted the National Health System (NHS) perspective^22^ ^,^ ^31^ ^,^ ^33^; and 1 study adopted both.^30^ A societal perspective was considered in 2 studies,^34^ ^,^ ^35^ and a national insurance perspective was considered in 1 study.^37^

Direct costs included testing the source and exposed HCP as well as post-exposure medical visits and treatment (ie, prophylaxis). Costs to treat an occupational infection were included in 2 studies only, which reported annual treatment costs^32^ and lifetime medical costs^35^ for HBV (Int$3,600 and Int$31,306, respectively), HCV (Int$24,424 and Int$23,173, respectively), and HIV infection (Int$35,745 and Int$441,342, respectively). These were excluded from the calculation of average NSI costs.

Indirect costs included lost productivity (time off work) associated with the time required for reporting and receiving initial and follow-up treatment as well as exposure consequences (eg, absence due to emotional distress and anxiety), overhead, compensation, and litigation.

Considering the 11 studies that provided aggregate direct + indirect costs, the overall median of the means of costs for managing an NSI was Int$747 (mean of means, Int$861; range of means, Int$199– Int$1691) (Tables 2 and 4).

**TABLE 4:** Description of the Distribution of the Means of the Costs for Managing a Single Percutaneous Injury (2015 International US Dollars)

			Means of the Costs for Managing a Single NSI				
Approach	Type of Cost	No. of Studies	Median	Mean	SD	Min	Max
Data driven	Direct	5	173	521	610	48	1,516
(N=8)	Indirect	3	175	232	119	152	369
	Direct + indirect	6	656	831	630	199	1,691
Modeling	Direct	4	586	595	285	294	913
(N=6)	Indirect	3	322	346	59	303	413
	Direct + indirect	5	747	897	284	649	1,324
All (N=14)	Direct	9	425	554	467	48	1,516
	Indirect	6	322	286	117	152	413
	Direct + indirect	11	747	861	482	199	1,691

NOTE. NSI, needlestick and sharps injury.

The median of the means of the 9 studies that estimated disaggregated direct costs was Int$425 (mean, Int$554; range, Int$48–Int$1,516), and the median of the means of the 6 studies that estimated disaggregated indirect costs was Int$322 (mean, Int$286; range, Int$152–Int$413).

Considering the type of approach, modeling studies had higher disaggregated and aggregated costs than data-driven studies: direct cost ratio, 3.4:1; indirect cost ratio, 1.84:1; combined direct+indirect cost ratio, 1.13:1. Costs from data-driven studies, however, showed greater variability, with standard deviations more than twice those of modeling studies (Table 4).

When estimating direct and indirect NSI management costs, the included cost items differed among studies (Table 5). Regarding direct costs, while laboratory tests represented the greater proportion in all studies, tests performed on the source and exposed HCP varied greatly. In total, 12 studies provided an itemized serological test menu.^8^ ^,^ ^17^ ^,^ ^22^ ^,^ ^24^ ^,^ ^25^ ^,^ ^29^ ^–^ ^31^ ^,^ ^33^ ^,^ ^34^ ^,^ ^36^ ^,^ ^37^ In all studies, investigators screened the source for antibodies against HCV (HCV-Ab) and HIV (HIV-Ab). HBV screening was performed mainly using surface antigen (HBsAg),^8^ ^,^ ^24^ ^,^ ^25^ ^,^ ^29^ ^,^ ^30^ ^,^ ^31^ ^,^ ^34^ ^,^ ^36^ adding surface antibody (HBsAb)^22^ or core antibody (HBcAb)^33^ ^,^ ^37^ or through HBcAb alone.^17^

**TABLE 5:** Description of Cost Items Included in 14 Studies of Economic Analysis on Occupational Needlestick and Sharps Injuries Among Healthcare Personnel, 1997–2013

Direct costs						Indirect costs		Study (Reference)
Laboratory Tests	DrugsHIV-PEP	HBV Vaccine	HBIG	Subsequent Occupational Infection	Post-Exposure Counseling/visit	Lost Productivity	Others	
Detailed costs; decision algorithm not specified	AZT + 3TC + IDV (cost for a 4-week treatment)	Cost for 3 doses and booster dose; cost in hospital A includes blood test	Hospital A: cost per dose; hospital B: cost for 5 doses	Not included	Cost depending on simple, moderate, and extensive risk	Hospital A: average; hospital B: not included	Not included	33
Detailed costs; baseline screening for HBV of the source always performed, regardless of HCP vaccinal status; baseline HCP screening for HCV and HIV depending on the serostatus of the source	Not performed (no HIV exposures)	Not included (charge borne by NHS)	Cost/dose included in the “post-exposure counselling/visit” cost	Not included	Cost/min for physician, nurse and support staff	Cost/min	Overheads	37
Non-detailed costs; decision algorithm not specified; baseline screening of the source and HCP for HBV, HCV and HIV is always performed.	AZT or ddI + PI; costs include an average of 14 days of PEP and tests to detect drug toxicity and a week off work	Not included	Not performed	Not included	Not included	Only for those who took HIV-PEP, evaluated using the average gross salary for nurses and included in PEP cost	Not included	17
Detailed costs; source screening for HBV depending on the HBV serostatus of the HCP; baseline HCP screening for HCV and HIV depending on the serostatus of the source	Average cost. AZT/3TC + NFV (1 case switched to d4T + ddI + IDV)	Not performed (no HBV exposure)		No subsequent occupational infection	Cost/injury for physician, nurse, consultant, and support staff	Cost/injury	Not included	36
Detailed costs; decision algorithm not specified	AZT alone regimen + 1 multidrug regimen	Not included	Not included	Not included	Cost/injury for employee health department, primary care physician, and consultant visit	Average	Not included	34
Detailed costs; baseline screening for HBV of the source is always performed, regardless of HCP vaccinal status. Baseline HCP screening for HCV and HIV depending on the serostatus of the source	AZT + 3TC + IDV (cost includes tests to detect drug toxicity)	Not included (charge borne by NHS)	Cost/dose included in the “post-exposure counselling/visit” cost	Not included	Cost/min for physician, nurse, and support staff	Cost/min for physician, nurse and support staff	Overheads	22
Detailed costs; baseline screening of source and HCP and FU not explicitly reported, but deriving from available national guidelines	AZT/3TC+ LPV/r (cost for a 4-wk treatment)	Cost/dose	Cost/2 doses	Only 24-wk treatment for HCV infection	Cost/min for employee health department and physician visit	Cost/visit for exposed doctor and nurse	Not included	30
Detailed costs; baseline screening of the HCP for HCV and HIV is always performed regardless of the serostatus of the source; baseline screening for HBV, HCV and HIV of the source is performed if unknown	AZT + 3TC + NFV (cost includes tests to detect drug toxicity)	Not included (charge borne by NHS)	Cost/dose	Not included	Cost/injury for physician and nurse	Not included	Not included	29
Non-detailed costs; decision algorithm not specified	Regimen not specified. Cost included test and HIV-PEP	Not specified; cost included test and HBV-PEP		HBV/HCV/HIV lifetime medical cost	Not included	Cost depending on intensity of follow-up, and subsequent chronic HBV, chronic or acute HCV, or HIV infection	Not included	35
Average cost; decision algorithm not specified	Regimen not specified; average cost	Cost included both HBV and HIV-PEP (no source patients mono-infected with HBV)		Not included	Mean cost and range for initial evaluation, exposure management, and follow-up visits	Mean cost and range for initial evaluation, exposure management, and follow-up	Not included	24
Detailed costs; baseline HCP screening for HBV, HCV, and HIV, depending on the serostatus of the source	Regimen not specified; cost for a 4-wk treatment	Cost/dose	Not included	Not included	Cost/visit for nurse, infectious disease specialist, and psychiatric consultant	Not included	Not included	31
Detailed costs; baseline HCP screening for HBV, HCV, and HIV, depending on the serostatus of the source	Regimen not explicitly reported, but deriving from available national guidelines; total cost for all exposures	Not performed (no HBV exposure in unvaccinated HCP)		Not included	Total cost for initial evaluation, exposure management, follow-up, and emergency department visits	Not included	Not included	8
Non-detailed costs; decision algorithm not specified	TDF/FTC + LPV/r for 4 weeks; average cost for low- and high-risk NSIs, including both HBV- and HIV-PEP	4 doses for unvaccinated and 1 booster dose for vaccinated HCP; average cost for low- and high-risk NSIs, including both HBV- and HIV-PEP	Not included	HBV/HCV/HIV subsequent infection	Average cost for low- and high-risk NSIs	Average cost for low- and high-risk NSIs	Compensation and litigation	32
Average costs; no decision algorithm	Regimen not specified; average cost/injury	Average cost/injury (cost included both HBV vaccine and HBIG)		Not included	Average cost/injury for medical consultations and surgical treatment	No lost productivity among the exposed HCP	Not included	25

NOTE. 3TC, lamivudine; AZT, zidovudine; d4T, stavudine; ddI, didanosine; FU, follow-up; HBIG, hepatitis B immune globulin; HBV, hepatitis B virus; HCV, hepatitis C virus; HCP, healthcare personnel; HIV, human immunodeficiency virus; IDV, indinavir; LPV/r, lopinavir/ritonavir; NFV, nelfinavir; NHS, National Health Service; NSI, needlestick and sharps injury; PEP, post-exposure prophylaxis; PI: protease inhibitor; TDF/FTC: tenofovir/emtricitabine.

Regarding HCP, in 4 studies,^17^ ^,^ ^29^ ^,^ ^30^ ^,^ ^33^ HCV-Ab and HIV-Ab were always performed; in 6 studies,^8^ ^,^ ^22^ ^,^ ^24^ ^,^ ^31^ ^,^ ^36^ ^,^ ^37^ based on the source serostatus; and in 2 studies,^25^ ^,^ ^34^ the tests were based on HCP anxiety or other factor. HBV baseline screening varied; HBsAb was performed in vaccinated HCP regardless of the source,^8^ in case of a HBsAg-positive source,^29^ or following exposure to a positive or unknown source.^22^ ^,^ ^24^ ^,^ ^30^ ^,^ ^37^ In 1 study, unvaccinated HCP, or vaccinated HCP with unknown or negative response, were tested for HBsAg, HBcAb, and HBsAb when exposed to an HBsAg-positive or unknown source.^36^ In another study, all HCP were tested only for HBcAb.^17^ Finally, in 4 studies, HBV markers (usually HBsAg and Ab) were performed regardless of the HCP vaccinal status or source serostatus.^25^ ^,^ ^31^ ^,^ ^33^ ^,^ ^34^

Furthermore, in a single study, the source and HCP were also screened for syphilis, and in some cases, the source was screened for hepatitis A virus. These tests account respectively for 13.4% and 1.7% of the total tests performed in the study period.^25^

The antiretroviral HIV PEP regimen was specified in 8 of 13 studies. In 6 studies conducted between 1990 and 2003, a zidovudine-based regimen was adopted alone (in 1 study)^34^ or in combination with lamivudine, including a protease inhibitor according to risk (indinavir in 3 studies^17^ ^,^ ^22^ ^,^ ^33^; nelfinavir in 2 studies^29^ ^,^ ^36^). In 2 more recent modeling studies (2006 and 2013), a protease inhibitor-based regimen with lopinavir/ritonavir was considered, adding zidovudine/lamivudine^30^ or tenofovir/emtricitabine^32^ (Table 5).

Finally, administration of hepatitis B immune globulin (HBIG) and HBV vaccination were included in the direct costs of an NSI in eight^22^ ^,^ ^24^ ^,^ ^25^ ^,^ ^29^ ^,^ ^30^ ^,^ ^33^ ^,^ ^35^ ^,^ ^37^ and seven^24^ ^,^ ^25^ ^,^ ^30^ ^,^ ^33^ ^,^ ^35^ studies, respectively (Table 5); in a single hospital study, HBV vaccination was administered but not included in the direct costs because the charges were borne by the NHS.^29^

Regarding indirect costs, 10 studies^17^ ^,^ ^22^ ^,^ ^24^ ^,^ ^30^ ^,^ ^32^ ^-^ ^37^ included lost productivity of an exposed HCP (eg, for initial reporting and treatment, follow-up appointments, and additional work time missed due to PEP side effects); 1 study^32^ included also compensation and litigation costs (Table 5).

### Quality of the Studies

The quality of the studies is presented in Table 6. For all investigations, the research question (item #1), economic importance of the research question (#2), viewpoints of the analysis (#3), primary outcome measures for the economic evaluation (#11), time horizon of costs and benefits (#22), and conclusions from the data reported (#34) were stated.

**TABLE 6:** Quality Results of the Selected Studies of Economic Analysis on Occupational Needlestick and Sharps Injuries Among Healthcare Personnel, 1997–2013

Author	Year	Total Score	Maximum Score^a^	Quality %
Jagger J^33^	1998	74	107	69
Solano Bernad VM^37^	1998	96	113	85
Roudot-Thoraval F^17^	1999	62	107	58
Nidegger D^36^	2003	63	79	80
Lee WC^34^	2005	72	85	85
Solano VM^22^	2005	72	89	81
Cazzaniga S^30^	2006	93	116	80
Armadans Gil L^29^	2006	107	110	97
Leigh JP^35^	2007	82	85	96
O’Malley EM^24^	2007	80	107	75
Glenngård AH^31^	2009	66	107	62
Fica CA^8^	2010	71	91	78
Hanmore E^32^	2013	106	113	94
Oh HS^25^	2013	65	79	82

a Maximum score represents the expected score if the study was conducted with optimal practices. Different Maximum scores were shown because different study designs were reviewed according to Drummond’s scale.^28^

Three studies presented the incremental analysis (#31), provided details of the design and results of effectiveness (#9), and used a synthesis method or meta-analysis of estimates (#10): the data-driven study by Armadans Gil, which had the highest quality score (97%),^29^ and 2 modeling studies, Leigh (96%)^35^ and Hanmore (94%).^32^ These 3 studies reported very different values of aggregate direct + indirect costs, with means of Int$418, Int$747, and Int$1,049, respectively.

## DISCUSSION

The overall aggregate direct + indirect costs for managing an NSI ranged from Int$650 to Int$750, considering the median and mean value of the mean costs borne by the hospital or NHS to manage exposures with different scenarios. These values were derived from both modeling and data-driven studies conducted across different countries and continents over a period of approximately 20 years.

Indirect costs are relatively consistent between studies; they mostly refer to lost productivity, which is usually calculated in minutes spent in baseline and follow-up visits by the exposed HCP and more rarely on days of staff absence; the median and mean values of the mean indirect costs ranged between Int$175 and Int$350.

Direct costs vary. The wide range of possible scenarios regarding the infectivity of the source, the susceptibility of the exposed HCP, and the post-exposure diagnostic and prophylactic protocol account for the differences in the average direct costs within and between considered studies, particularly when analyzing studies using a different approach. In modeling studies, the source and exposed HCP are tested according to an optimized protocol. Prophylaxes are provided to all susceptible HCP, and those protocols are always considered to have been completed. All exposed HCP attend follow-up visits and testing. In real life, HCP anxiety can influence the choice of baseline and follow-up testing, or the source situation may be more complicated (eg, multiple possible sources, need for supplemental, confirmatory testing, etc.). Acceptance, adherence, and completion rate of prophylactic treatments may be suboptimal; regimens may vary and cause adverse reactions requiring additional interventions; and compliance with follow-up protocols may differ.

Moreover, regardless of the approach, treatment costs vary between studies: the standard antiretroviral PEP regimen changed significantly over time, including different combinations and newer, more tolerable and expensive drugs in recent studies. Variations in HBIG dosage among protocols increased costs up to 4-fold, and diagnostic tests evolved over time. Two recent modeling studies considering direct and indirect costs and using newer antiretrovirals had a median cost of Int$1,187 (range, Int$1,049–Int$1,324)^30^ ^,^ ^32^; however, too few recent studies are available to define a real and current overall cost.

Notwithstanding all the observed differences, comparisons between studies also identified significant similarities. Therefore, considering the wide range of possible situations, settings, and behaviors covered in selected studies, this overall amount (including direct and indirect costs) should represent a reliable estimate of the actual average cost for managing an NSI. However, the wide range of observed values should be taken into due account to avoid under- or overestimating the economic costs of managing these incidents or the potential savings resulting from their prevention.

A cautious interpretation of these findings is warranted. The literature search may be incomplete; moreover, different periods, countries, and study designs were compared. Part of the heterogeneity due to the approach and type of estimated costs was controlled by stratifying the studies. Furthermore, weighted means were calculated where disaggregated costs were available. However, this was not possible for all the studies considered. The effect of this heterogeneity is evident in the high variability (SD of the mean and width of the range) of the estimates. In addition, Drummond’s checklist was designed to include all possible aspects concerning economic evaluations, but in this review, different study designs were compared, and it was not possible to apply all items to all articles. To eliminate disparities between the studies, a quality score was calculated based on the items considered appropriate for each study.

Despite these issues, this review confirms that the economic impact of managing NSIs is not negligible, and our analysis provides clues about where to reduce costs.

Protocols should be optimized based on the source; HCP vaccination should increase, and the response should be appropriately evaluated post-vaccination and readily available in case of injury. This recommendation is clearly demonstrated in the high-quality study by Armadans Gil et al,^29^ where most exposures involved vaccinated HCP exposed to known sources who tested negative for HCV and HIV; the mean NSI management cost (direct + indirect) was Int$418.

Additional strategies to reduce costs could include the following: using rapid HIV tests to assess the source serostatus, thus avoiding unnecessary PEP, tests, and anxiety for the exposed HCP,^38^ and implementing tests for the early detection of bloodborne infections, which can shorten follow-up periods (eg, HIV-Ab/Ag test) and allow for early diagnosis at a lower cost (eg, HCV core-antigen testing replacing HCV-RNA).

Even if none of the retrieved studies included HCV-RNA testing in post-exposure protocols, it is recommended as alternative strategy in the United States and is strongly supported in a cost-effectiveness analysis. HCV-RNA testing at 1 month, at an average cost of Int$242 per exposure, leads to earlier detection of HCV transmission and lowers the risk of progression to chronic hepatitis.^39^ Consequently, NSI management costs could also increase, and even more in the future, if direct-acting antivirals for hepatitis C are used in PEP, which would add a significant cost to treating these frequent events.

Are these costs truly representative of this issue? The costs of treating an occupational infection were not included in the calculation of average NSI costs, nor were those of litigation and compensation. Bloodborne infections other than HBV, HCV, or HIV were not considered, though many different pathogens can be, and have been, occasionally transmitted through an NSI. Most importantly, intangible costs arising from an NSI were not evaluated. The single willingness-to-pay analysis identified in the literature reported that the high median amount HCP were willing to pay to avoid a sharps-related injury were Int$828 in the base case and Int$1,237 when exposed to HIV or HCV. This finding suggests that the costs of intangible aspects of HCP injuries, such as anxiety and distress, could equal costs associated with the medical evaluation of these injuries.^40^ However, in this study, the median time from injury to interview was 3 days. Thus, the interviewed subjects had not yet experienced the impact of being at risk of developing a bloodborne infection or the effects on personal and family life, sexual relationships, reproductive plans, breastfeeding, or professional expectations, which would increase the amounts reported above.

In conclusion, NSIs generate significant direct, indirect, potential, and intangible costs. While the costs for their prevention may seem high in the beginning, ultimately they prove to be the opposite. The economic expenditures directed toward enhancing interventions to prevent occupational exposures and infections, including provision of SEDs, will likely decrease over time, while enhancing HCP perception of their own value and affecting the quality of the care they provide. As highlighted in the preamble of the EU Directive, “Health and safety of workers is paramount and is closely linked to the health of patients.”
